# Long-term trends in cancer incidence and mortality among U.S. children and adolescents: a SEER database analysis from 1975 to 2018

**DOI:** 10.3389/fped.2024.1357093

**Published:** 2024-07-05

**Authors:** Xiao-Wei Tang, Jiao Jiang, Shu Huang, Xiao-Min Shi, Huan Xu, Jia Xu, Jie-Yu Peng, Wei Zhang, Lei Shi, Xiao-Lin Zhong, Min Kang, Mu-Han Lü

**Affiliations:** ^1^ Department of Gastroenterology, the Affiliated Hospital of Southwest Medical University, Luzhou, China; ^2^ Nuclear Medicine and Molecular Imaging Key Laboratory of Sichuan Province, The Affiliated Hospital of Southwest Medical University, Luzhou, China; ^3^ Department of Gastroenterology, Lianshui County People’ Hospital, Huaian, China; ^4^ Department of Gastroenterology, Lianshui People’ Hospital of Kangda College Affiliated to Nanjing Medical University, Huaian, China

**Keywords:** incidence, pediatric cancer, cancer death rates, children and adolescents, trend

## Abstract

**Background:**

Childhood and adolescent cancer represent a significant health burden in the United States. Current and precise epidemiological data are crucial to develop effective cancer control plans and ultimately reduce the burden of childhood and adolescent cancer.

**Methods:**

We analyzed data obtained from cancer registries in the National Cancer Institute's Surveillance, Epidemiology, and End Results Program. Age-standardized incidence and death rates, assessed using joinpoint analysis, were quantified as annual percentage changes (APC) and average percentage changes (AAPC).

**Results:**

The overall cancer incidence rate in 2008–2018 was 187.9 per 1,000,000 persons. Cancer incidence rates demonstrated a sustained upward trend, with an APC of 0.8 from 1975 to 2018. Incidence rates during 2008–2018 remained stable among non-Hispanic Black children but increased among other racial and ethnic groups. Leukemias, central nervous system tumors, and lymphomas were the most common cancer groups for patients aged 0–19 years. Cancer death rates decreased among children [AAPC, −1.3 (95% CI, −1.5 to −1.1)] during 2009–2019, while were stable among adolescents during that period.

**Conclusions:**

In this study, we analyzed cancer incidence and mortality rates and trends in children aged 0–19 years in the United States. Our findings revealed an overall increase in cancer incidence rates among children and adolescents, accompanied by a decline in cancer mortality rates over time. These rates and trends varied by age, sex, and particularly race and ethnicity, highlighting the significance of comprehending and addressing disparities and ultimately reducing the disease burden of childhood and adolescent cancer.

## Introduction

Cancer is a formidable health challenge that affects individuals of all ages, even in children (ages 0–14 years) and adolescents (ages 15–19 years) (1). Childhood cancer encompasses a broad range of malignancies that can arise in various organs and tissues, which differ from the cancers found in adults (2). While childhood and adolescent cancer is relatively rare compared to cancer in adults, it remains a significant concern due to its profound impact on the lives of young patients and their families.

In the United States, childhood and adolescent cancer represents a significant health burden. Cancer is the leading disease-related cause of death in this age group (3). In 2023, it was estimated that 9,910 children and 5,280 adolescents received a diagnosis of cancer, while approximately 1,040 children and adolescents were expected to have died from the disease (4). On the other hand, when considering the disability burden caused by childhood and adolescent cancer, a study used disability-adjusted life-years (DALYs) as a metric and reported that childhood and adolescent cancer resulted in a substantial DALY burden, highlighting the long-term impact of the disease on quality of life and overall well-being (5).

Understanding accurate epidemiological data in this vulnerable population is crucial for health policy prioritization, developing cancer control plans, and ultimately reducing the burden of cancer-related morbidity and mortality (2). Previous studies have endeavored to determine the epidemiological characteristics in children and adolescents in the United States. Several of these studies described that at a specific time or evaluated the trends for limited periods (6–8), while others focused on specific cancer types (9–11). Research investigating the long-term trends in incidence and mortality rates by age, sex, and race and ethnicity among children and adolescents is lacking. Hence, this study aimed to expand on these studies to describe cancer incidence and mortality rates and trends in a more detailed way. By using data obtained from cancer registries in the National Cancer Institute's Surveillance, Epidemiology, and End Results (SEER) Program, we evaluated the long-term trends and cross-sectional incidence and mortality rates for children and adolescent cancer.

## Methods

### Data source

Population-based cancer incidence and mortality data were obtained from cancer registries in the SEER Program. The SEER program was established in 1973 (SEER 9 registry) and has undergone 2 major expansions (SEER 13 in 1992 and SEER 18 in 2000) to incorporate additional areas. SEER 18 covers about 28% of the U.S. population. Hence, in order to enhance the representativeness of this study, the SEER 9 database was used to cover data from 1975 to 1991, the SEER 13 database to cover data from 1992 to 1999, and the SEER 18 database to cover data from 2000 to 2018.

### Case definition

For the analysis of cancer incidence rates and trends, the study included patients aged 0–19 years diagnosed with a primary malignant neoplasm in the United States between 1975 and 2018. This age group encompassed children (aged 0–14 years) and adolescents (aged 15–19 years). For the analysis of cancer mortality rates and trends, the study included all individuals within this age population who died of malignant cancers between 1975 and 2019. Cancer diagnoses were categorized based on histology and primary site, according to the third edition of the International Classification of Childhood Cancer (ICCC-3). Cause of death was coded according to the International Classification of Diseases (ICD) categories, specifically ICD-8 to ICD-10 in this study. Rates and trends were presented by age, sex, and race and ethnicity. Race and ethnicity were divided into the following 5 mutually exclusive racial/ethnic groups: non-Hispanic White, non-Hispanic Black, non-Hispanic American Indian or Alaska Native (AI/AN), non-Hispanic Asian or Pacific Islander (API), and Hispanic (all races). Notably, given that rates categorized by expanded race (white, black, AI/AN, and API) and Hispanic ethnicity have been available since 1992, trends by racial and ethnic group have been calculated since 1992. Mortality rates and trends by ICD group were not displayed for non-Hispanic AI/AN and non-Hispanic API due to a small number of cases.

### Statistical analysis

We used SEER*Stat software (version 8.4.1, developed by the National Cancer Institute and Information Management Services) to calculate the incidence and mortality rates (12). Rates were expressed per 1,000,000 persons and were age-adjusted to the 2000 US standard population (19 age groups, Census P25–1,130). Cross-sectional rates were calculated for incidence from 2008 to 2018 and for mortality from 2009 to 2019. Rates were considered unreliable and therefore not reported if there were fewer than 16 cases or deaths during the specified time period. Death rates were restricted to the top 12 causes of cancer death. Temporal trends in incidence (1975–2018) and death (1975–2019) rates were quantified by using annual percent change (APC) and average APC (AAPC), which is a summary representation of the trend over a specific time interval. The corresponding 95% confidence intervals (95% CI) were calculated using the parametric method in Joinpoint Regression Program, version 4.9.1.0. Trends were deemed unreliable and not calculated if there were fewer than 10 cases or deaths in any 1 calendar year (13). Statistical significance was determined using a *t*-test for the APC. For AAPC, a *t*-test was used when it lay within the last segment; otherwise, a *z*-test was used. Two-sided *P* < .05 was considered statistically significant.

## Results

### Cancer incidence by age, sex, and race

**Figure 1:**
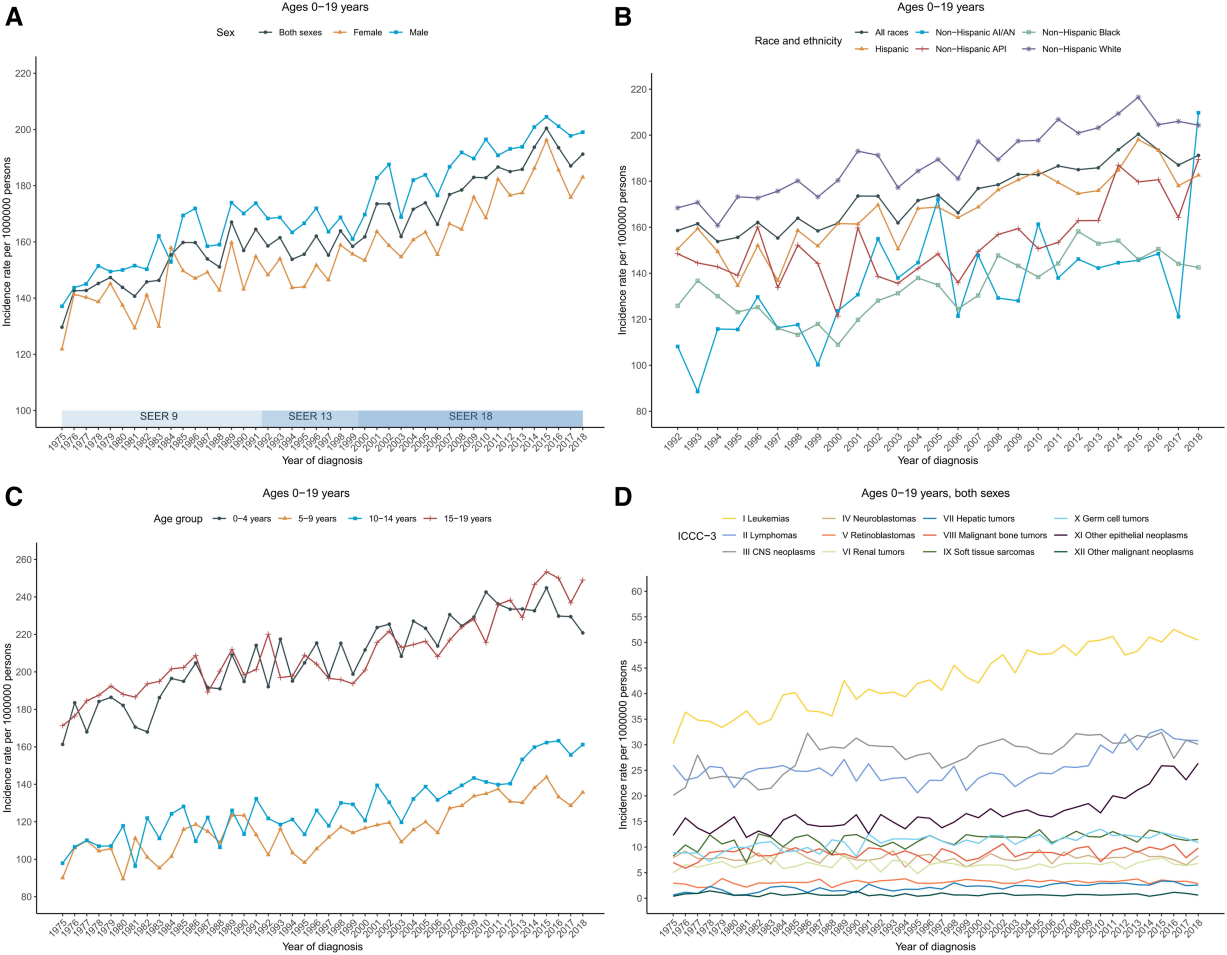
Cancer incidence trends for children and adolescents (ages 0–19 years) in the United States. (**A**) Annual age-adjusted incidence of all cancers by sex (1975–2018). (**B**) Cancer rates by race and ethnicity. (**C**) Cancer rates by age at diagnosis. (**D**) Cancer rates by the International Classification of Childhood Cancer, third edition (ICCC-3) group. Rates were per 1,000,000 persons, age-standardized to the 2000 US standard population (19 age groups, Census P25–1,130). The ICCC-3 is displayed by abbreviated title. SEER, Surveillance, Epidemiology, and End Results Program; AI/AN, American Indian and Alaska Native; and API, Asian and Pacific Islander.

The annual age-adjusted cancer incidence rate in patients aged 0–19 years was 129.6 (per 1,000,000 persons) in 1975 and increased to 191.2 by 2018. The overall cancer incidence rate in 2008–2018 was 187.9 and was higher in males (196.2) than in females (179.2). Cancer incidence rates over time showed an increasing long-term trend, with an APC of 0.8 during 1975–2018. Among boys, rates also increased with an APC of 0.8 during 1975–2018; among girls, rates increased from 1975 to 2015 with the steepest rise occurring between 2006 and 2015 [APC, 1.8 (95% CI, 1.0–2.7)], and then became stable during 2015–2018 (Figure 1A; Supplementary Table S1).

By racial/ethnic group, the overall incidence rates in 2008–2018 were highest in non-Hispanic White population (203.1), followed by Hispanic (182.6), non-Hispanic API (168.2), non-Hispanic Black (147.3), and non-Hispanic AI/AN (146.4). Trends in cancer incidence rate varied by race and ethnicity. Incidence rates during 2008–2018 increased among non-Hispanic White [AAPC, 0.9 (95% CI, 0.7–1.1)], non-Hispanic API [AAPC, 2.0 (95% CI, 1.2–2.8)], non-Hispanic AI/AN [AAPC, 1.4 (95% CI, 0.7–2.2)], and Hispanic populations [AAPC, 1.0 (95% CI, 0.8–1.3)], while remaining stable among non-Hispanic Black population (Figure 1B; Supplementary Table S1).

Incidence rates and trends also varied by age group. Adolescents (aged 15–19 years) had the highest average annual incidence rate in 2008–2018 (236.7), followed by children aged 0–4 years (232.5), 10–14 years (150.8), and 5–9 years (134.1). In addition, rate in children (aged 0–14 years) was 171.5 in 2008–2018. Rates in children aged 0–4 years and 5–9 years were stable between 2008 and 2018. Specifically, rates in children aged 0–4 years increased from 1975 to 2015 with an APC of 0.8 before decreasing insignificantly from 2015 to 2018; rates in children aged 5–9 years increased from 1975 to 2006 with an APC of 0.5 and then became stable since 2006. However, rates have been increasing for children aged 10–14 years since 1975, with an AAPC of 1.8 (95% CI, 1.1–2.4) during the period 2008–2018. Similarly, for children aged 14–19 years, rates have been increasing with an AAPC of 1.3 (95% CI, 0.8–1.8) during the same period (Figure 1C; Supplementary Table S1).

### Cancer incidence by ICCC-3 cancer group

**Figure 2:**
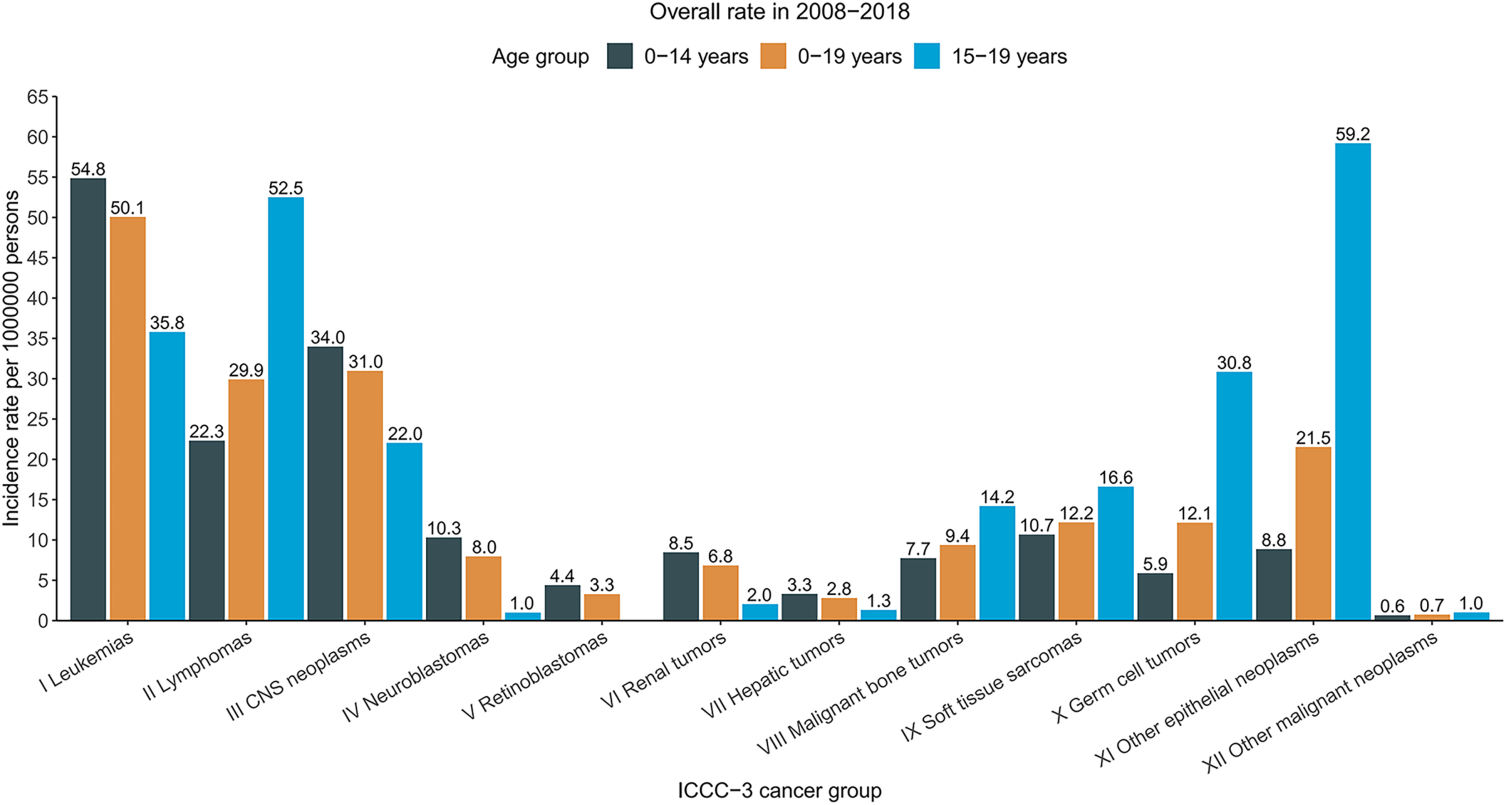
Cancer incidence rates of children, adolescents, and children and adolescents in the United States, 2008–2018, by the international classification of childhood cancer, third edition (ICCC-3) group. Rates were per 1000000 persons, age-standardized to the 2000 US standard population (19 age groups, Census P25–1,130). The ICCC-3 is displayed by abbreviated title.

Long-term trends in cancer incidence rates by the ICCC-3 cancer group were presented in Figure 1D. Over the 2008–2018 period, rates increased for central nervous system (CNS) tumors [AAPC, 0.3 (95% CI, 0.0–0.6)], hepatic tumors [AAPC, 1.9 (95% CI, 1.3–2.4)], malignant bone tumors [AAPC, 0.3 (95% CI, 0.0–0.6)], soft tissue and other extraosseous sarcomas [AAPC, 0.4 (95% CI, 0.1–0.6)], germ cell neoplasms [AAPC, 0.7 (95% CI, 0.4–0.9)], and Other malignant epithelial neoplasms and malignant melanomas [AAPC, 4.6 (95% CI, 3.4–5.8)] among patients aged 0–19 years with both sexes combined. Meanwhile, rates remained stable for leukemias, lymphomas, neuroblastomas, retinoblastomas, renal tumors, and other unspecified malignant neoplasms. Incidence trends differed slightly between males and females. For male patients aged 0–19 years, rates for leukemias increased 1.0% per year while rates for CNS tumors remained stable over the same period; for female patients, rates increased for leukemias (AAPC, 1.1), lymphomas (AAPC, 2.0), while rates for malignant bone tumors and germ cell neoplasms remained stable (Supplementary Table S1). Leukemias, central nervous system (CNS) tumors, and lymphomas were the most common cancer groups for children (aged 0–14 years, 54.8, 34.0, and 22.3, respectively) and for patients aged 0–19 years (50.1, 31.0, and 29.9, respectively), whereas other malignant epithelial neoplasms and malignant melanomas, lymphomas, and leukemias were the most common cancer types among adolescents (59.2, 52.5, and 35.8, respectively). The detailed overall cancer incidence rates in 2008–2018 stratified by age group were presented according to ICCC-3 group in Figure 2.

Table 1 presented the overall cancer incidence rates and trends in 2008–2018 for ICCC-3 cancer type by race and ethnicity. Rates by ICCC-3 group were not calculated for non-Hispanic AI/AN population because of few cases. Rates for females in every racial/ethnic group all increased, and the greatest increase was observed in non-Hispanic API population with an AAPC of 2.3 (95% CI, 1.3–3.2); rates for males increased in almost all racial/ethnic groups except for non-Hispanic AI/AN, which was stable during that period. The highest total incidence rate for leukemias was in Hispanic population (61.1). Rates for leukemias increased among all racial/ethnic groups (non-Hispanic White, non-Hispanic Black, non-Hispanic API, and Hispanic), with the largest increase of rates for acute myeloid leukemia (AML) in non-Hispanic Black population [AAPC, 1.4 (95% CI, 0.1–2.7)]. The highest rate for total lymphomas was in non-Hispanic White population (33.1). Rates for total lymphomas increased among all racial/ethnic groups, with the largest increase of rates in non-Hispanic API population [AAPC, 3.2 (95% CI, 2.4–3.9)]. Moreover, non-Hodgkin lymphomas (except Burkitt lymphoma) diagnosed in non-Hispanic Black population were the subgroup of lymphomas with the largest increase of rates [AAPC, 2.5 (95% CI, 1.3–3.7)]. Rates for CNS tumors increased among non-Hispanic White, non-Hispanic Black, and Hispanic populations. The largest rate was 38.4 in non-Hispanic White population. Astrocytoma was the most common subtype of CNS tumors among all racial/ethnic groups with the highest incidence rate of 20.6 in non-Hispanic White population. The highest rate for renal tumors was 9.1 in non-Hispanic Black population. Rates for hepatic tumors, characterized by hepatoblastoma and mesenchymal tumors of liver increased significantly among non-Hispanic White and Hispanic populations (AAPC for total hepatic tumors, 2.0 and 1.9, respectively), while the number of cases was still rare. The highest rate for soft tissue and other extraosseous sarcomas was 13.8 in non-Hispanic Black population. Germ cell neoplasms, specifically malignant gonadal germ cell tumors, were most common in Hispanic population and continued to increase with an AAPC of 1.6 (95% CI, 0.6–2.7) between 2008 and 2018. Rates for thyroid carcinomas increased significantly among all racial/ethnic groups, with the highest rate of 12.0 in non-Hispanic White and with the largest AAPC of 7.0 in non-Hispanic API population. Whereas the rate in non-Hispanic Black population (3.7) was rather small compared with other racial/ethnic groups. Rates for other unspecified carcinomas increased significantly among non-Hispanic White (AAPC, 8.9) and Hispanic populations (AAPC, 10.6).

**Table 1:** Age-standardized incidence rates and fixed-interval trends (2008–2018) for the ICCC-3 cancer group by racial/ethnic group, ages 0–19 years.

Sex and cancer site or type	All races/ethnicities			Non–Hispanic White			Non–Hispanic Black			Non–Hispanic API			Non–Hispanic AI/AN			Hispanic		
	Rate	AAPC^a^ (95% CI)	*P*	Rate	AAPC (95% CI)	*P*	Rate	AAPC (95% CI)	*P*	Rate	AAPC (95% CI)	*P*	Rate	AAPC (95% CI)	*P*	Rate	AAPC (95% CI)	*P*
All sites																		
Males	196.2	0.8 (0.7 to 0.9)	<.001	211.1	0.8 (0.6 to 1.0)	<.001	151.9	0.9 (0.4 to 1.4)	<.001	176.0	1.1 (0.6 to 1.5)	<.001	155.9	1.0 (–0.2 to 2.2)	0.10	193.4	1.0 (0.7 to 1.3)	<.001
Females	179.2	0.7 (–0.5 to 1.9)	.28	194.7	1.0 (0.8 to 1.3)	<.001	142.5	1.2 (0.7 to 1.6)	<.001	160.1	2.3 (1.3 to 3.2)	<.001	136.7	1.8 (0.4 to 3.2)	.01	171.4	1.1 (0.8 to 1.4)	<.001
ICCC–3 cancer group																		
I Leukemias, myeloproliferative diseases, and myelodysplastic diseases	50.1	0.4 (–0.2 to 1.1)	.20	47.1	0.7 (0.3 to 1.0)	<.001	31.8	1.3 (0.7 to 1.8)	<.001	48.8	0.8 (0.2 to 1.4)	.007	45.5	–	–	61.1	0.8 (0.5 to 1.1)	<.001
Ia Lymphoid leukemias	36.4	1.0 (0.8 to 1.1)	<.001	34.4	0.4 (0.1 to 0.8)	.02	18.3	0.7 (–0.1 to 1.5)	.07	32.7	0.6 (0.0 to 1.2)	.04	31.2	–	–	47.2	0.8 (0.4 to 1.1)	<.001
Ib Acute myeloid leukemias	8.8	1.0 (0.7 to 1.2)	<.001	8.1	0.3 (–0.2 to 0.9)	.19	8.4	1.4 (0.1 to 2.7)	.03	10.9	0.4 (–0.9 to 1.7)	.55	8.9	–	–	9.1	0.2 (–0.6 to 0.9)	.64
Ic Chronic myeloproliferative diseases	2.3	1.6 (0.2 to 3.0)	.03	2.3	–	–	2.4	–	–	2.8	–	–	–	–	–	1.8	–	–
Id Myelodysplastic syndrome and other myeloproliferative diseases	1.6	–	–	1.3	–	–	2.0	–	–	1.6	–	–	–	–	–	1.6	–	–
Ie Unspecified and other specified leukemias	1.1	1.4 (–0.5 to 3.3)	.16	1.0	–	–	0.8	–	–	0.8	–	–	–	–	–	1.4	–	–
II Lymphomas and reticuloendothelial neoplasms	29.9	1.4 (–0.3 to 3.2)	.10	33.1	2.4 (1.6 to 3.3)	<.001	26.4	2.0 (1.1 to 2.8)	<.001	27.5	3.2 (2.4 to 3.9)	<.001	13.9	–	–	26.3	1.7 (1.1 to 2.4)	<.001
IIa Hodgkin lymphomas	12.1	–0.5 (–0.7 to –0.3)	<.001	14.4	0.0 (–0.5 to 0.6)	.98	11.3	0.8 (–0.4 to 1.9)	.19	8.8	2.0 (0.4 to 3.6)	.01	–	–	–	9.9	–0.6 (–1.4 to 0.1)	.09
IIb Non–Hodgkin lymphomas (except Burkitt lymphoma)	10.5	1.9 (–1.1 to 4.9)	.22	10.6	1.7 (1.1 to 2.3)	<.001	11.0	2.5 (1.3 to 3.7)	<.001	11.5	2.4 (0.7 to 4.2)	.006	–	–	–	9.6	2.4 (1.7 to 3.2)	<.001
IIc Burkitt lymphoma	2.5	0.7 (0.2 to 1.2)	.01	3.3	0.4 (–0.8 to 1.6)	.52	1.8	–	–	2.2	–	–	–	–	–	1.7	–	–
IId Miscellaneous lymphoreticular neoplasms	4.3	14.2 (–0.7 to 31.4)	.06	4.6	–		1.7	–	–	4.6	–	–	–	–	–	4.7	–	–
IIe Unspecified lymphomas	0.4	–	–	0.3	–	–	0.6	–	–	–	–	–	–	–	–	0.4	–	–
III CNS and miscellaneous intracranial and intraspinal neoplasms	31.0	0.2 (0.0 to 0.5)	.04	38.4	0.8 (0.4 to 1.1)	<.001	26.8	0.8 (0.0 to 1.6)	.04	23.1	0.4 (–0.3 to 1.1)	.24	24.1	–		23.2	–1.1 (–1.9 to –0.1)	.02
IIIa Ependymomas and choroid plexus tumor	2.7	0.8 (0.3 to 1.3)	.001	2.9	0.8 (–0.2 to 1.8)	.12	2.4	–	–	2.3	–	–	–	–	–	2.7	0.5 (–0.9 to 1.9)	.47
IIIb Astrocytomas	15.9	–1.0 (–3.2 to 1.4)	.41	20.6	0.9 (0.4 to 1.5)	.003	13.3	1.0 (–0.1 to 2.2)	.07	10.6	0.6 (–0.7 to 1.9)	.36	14.5	–	–	11.0	0.0 (–0.6 to 0.7)	.90
IIIc Intracranial and intraspinal embryonal tumors	6.1	–0.7 (–1.5 to 0.0)	.06	7.3	–0.1 (–0.8 to 0.5)	.72	4.5	0.2 (–0.8 to 1.3)	.65	6.1	–	–	–	–	–	5.1	–1.8 (–2.9 to –0.7)	.002
IIId Other gliomas	5.3	0.4 (0.0 to 0.8)	.04	6.6	1.2 (0.5 to 2.0)	.003	5.2	0.0 (–1.6 to 1.6)	.99	3.5	–	–	–	–	–	3.7	0.5 (–1.1 to 2.1)	.53
IIIe Other specified intracranial and intraspinal neoplasms	0.6	–	–	0.6	–	–	0.9	–	–	–	–	–	–	–	–	0.6	–	–
IIIf Unspecified intracranial and intraspinal neoplasms	0.3	–	–	0.4	–	–	0.5	–	–	–	–	–	–	–	–	–	–	–
IV Neuroblastoma and other peripheral nervous cell tumors	8.0	0.0 (–0.3 to 0.3)	.82	9.8	0.2 (–0.5 to 1.0)	.52	7.8	–0.1 (–1.9 to 1.8)	.95	7.4	0.5 (–0.7 to 1.6)	.40	–	–	–	5.4	–0.1 (–0.8 to 0.6)	.76
IVa Neuroblastoma and ganglioneuroblastoma	7.7	0.0 (–0.3 to 0.3)	.77	9.5	0.2 (–0.6 to 1.0)	.59	7.4	–0.1 (–1.9 to 1.8)	.92	7.4	0.6 (–0.6 to 1.9)	.30	–	–	–	5.2	0.0 (–0.8 to 0.7)	.92
IVb Other peripheral nervous cell tumors	0.3	–	–	0.3	–	–	–	–	–	–	–	–	–	–	–	–	–	–
V Retinoblastoma	3.3	0.3 (–0.0 to 0.6)	.07	3.0	–0.3 (–1.2 to 0.7)	.55	3.5	–	–	3.6	–	–	–	–	–	3.4	0.0 (–1.1 to 1.2)	.95
VI Renal tumors	6.8	0.1 (–0.1 to 0.4)	.30	7.5	0.4 (–0.2 to 1.0)	.16	9.1	0.2 (–1.1 to 1.6)	.72	3.8	–	–	–	–	–	5.7	1.0 (–0.2 to 2.2)	.09
VIa Nephroblastoma and other non–epithelial renal tumors	6.1	0.0 (–0.3 to 0.3)	.81	6.8	0.2 (–0.5 to 0.9)	.54	7.9	0.3 (–1.0 to 0.7)	.62	3.1	–	–	–	–	–	5.2	0.7 (–0.4 to 1.9)	.19
VIb Renal carcinomas	0.7	–	–	0.7	–	–	1.2	–	–	–	–	–	–	–	–	0.4	–	–
VIc Unspecified malignant renal tumors	–	–	–	–	–	–	–	–	–	–	–	–	–	–	–	–	–	–
VII Hepatic tumors	2.8	1.9 (1.3 to 2.4)	<.001	2.7	2.0 (0.9 to 3.0)	.001	2.0	–	–	3.5	–	–	–	–	–	3.0	1.9 (0.1 to 3.8)	.04
VIIa Hepatoblastoma and mesenchymal tumors of liver	2.2	2.3 (1.7 to 2.9)	<.001	2.1	2.0 (0.8 to 3.2)	.003	1.5	–	–	2.9	–	–	–	–	–	2.4	1.8 (–0.1 to 3.7)	.06
VIIb Hepatic carcinomas	0.6	–	–	0.6	–	–	0.5	–	–	–	–	–	–	–	–	0.6	–	–
VIIc Unspecified malignant hepatic tumors	–	–	–	–	–	–	–	–	–	–	–	–	–	–	–	–	–	–
VIII Malignant bone tumors	9.4	0.3 (0.0 to 0.6)	.03	10.2	0.5 (–0.2 to 1.3)	.16	7.9	0.5 (–0.8 to 1.9）	.43	8.2	–0.2 (–1.8 to 1.4)	.82	–	–	–	8.9	0.3 (–0.6 to 1.2)	.48
VIIIa Osteosarcomas	5.4	0.6 (0.3 to 1.0)	.001	5.2	0.8 (0.1 to 1.6)	.03	6.2	0.7 (–0.9 to 2.2)	.40	4.8	–	–	–	–	–	5.5	0.1 (–1.0 to 1.2)	.88
VIIIb Chondrosarcomas	0.4	–		0.5	–	–	0.5	–	–	–	–	–	–	–	–	0.3	–	–
VIIIc Ewing tumor and related sarcomas of bone	2.9	–0.2 (–0.7 to 0.3)	.48	3.9	0.2 (–0.9 to 1.2)	.75	0.6	–	–	2.4	–	–	–	–	–	2.4	0.4 (–0.9 to 1.8)	.54
VIIId Other specified malignant bone tumors	0.4	–	–	0.5	–	–	–	–	–	–	–	–	–	–	–	0.4	–	–
VIIIe Unspecified malignant bone tumors	0.2	–	–	–	–	–	–	–	–	–	–	–	–	–	–	0.2	–	–
IX Soft tissue and other extraosseous sarcomas	12.2	0.4 (0.1 to 0.6)	.002	12.3	–0.2 (–0.8 to 0.3)	.35	13.8	0.2 (–0.8 to 1.2)	.70	10.3	0.3 (–1.2 to 1.8)	.68	11.5	–	–	11.4	–0.2 (–1.1 to 0.8)	.74
IXa Rhabdomyosarcomas	4.5	0.0 (–0.3 to 0.4)	.80	4.8	0.0 (–0.9 to 0.9)	.95	5.3	–0.7 (–2.1 to 0.7)	.30	3.9	–	–	–	–	–	3.8	0.1 (–1.2 to 1.4)	.91
IXb Fibrosarcomas, peripheral nerve sheath tumors and oth fibrous neoplasms	1.2	–0.7 (–1.3 to –0.0)	.04	1.2	–	–	1.4	–	–	0.9	–	–	–	–	–	1.1	–	–
IXc Kaposi sarcoma	–	–	–	–	–	–	–	–	–	–	–	–	–	–	–	–	–	–
IXd Other specified soft tissue sarcomas	5.0	0.9 (0.5 to 1.2)	<.001	4.8	–0.3 (–1.0 to 0.5)	.48	5.4	0.9 (–0.6 to 2.4)	.22	4.2	–	–	–	–	–	5.2	1.1 (–0.0 to 2.3)	.053
IXe Unspecified soft tissue sarcomas	1.4	0.8 (0.1 to 1.5)	.03	1.5	–	–	1.6	–	–	1.3	–	–	–	–	–	1.2	–	–
X Germ cell tumors, trophoblastic tumors, and neoplasms of gonads	12.1	0.7 (0.4 to 0.9)	<.001	11.0	–0.4 (–0.9 to 0.0)	.06	6.6	–0.3 (–1.6 to 1.0)	.63	13.0	–0.1 (–1.3 to 1.0)	.81	10.1	–	–	15.7	1.1 (0.3 to 1.9)	.01
Xa Intracranial and intraspinal germ cell tumors	1.9	1.2 (0.6 to 1.9)	<.001	1.7	–0.3 (–1.4 to 0.8)	.56	1.3	–	–	4.1	–	–	–	–	–	1.9	–	–
Xb Malignant extracranial and extragonadal germ cell tumors	1.4	–0.5 (–1.1 to –0.0)	.04	1.3	–1.2 (–2.4 to 0.0)	.051	1.3	–	–	2.2	–	–	–	–	–	1.4	–	–
Xc Malignant gonadal germ cell tumors	8.0	0.8 (0.5 to 1.1)	<.001	7.3	–0.5 (–1.2 to 0.2)	.14	3.4	–	–	5.9	0.6 (–2.0 to 0.8)	.38	7.6	–	–	11.6	1.6 (0.6 to 2.7)	.003
Xd Gonadal carcinomas	0.4	–	–	0.4	–	–	–	–	–	0.7	–	–	–	–	–	0.6	–	–
Xe Other and unspecified malignant gonadal tumors	0.3	–	–	0.2	–	–	0.5	–	–	–	–	–	–	–	–	0.3	–	–
XI Other malignant epithelial neoplasms and malignant melanomas	21.5	4.6 (3.4 to 5.8)	<.001	27.0	4.1 (3.1 to 5.2)	<.001	10.8	1.9 (0.7 to 3.2)	.004	18.3	5.1 (3.5 to 6.7)	<.001	16.8	–		17.7	6.4 (4.9 to 8.0)	<.001
XIa Adrenocortical carcinomas	0.3	–	–	0.3	–	–	–	–	–	–	–	–	–	–	–	0.3	–	–
XIb Thyroid carcinomas	10.2	5.3 (4.3 to 6.4)	<.001	12.0	3.9 (3.2 to 4.6)	<.001	3.7	4.4 (2.3 to 6.5)	<.001	11.1	7.0 (4.5 to 9.7)	<.001	–	–	–	9.8	4.8 (3.6 to 6.1)	<.001
XIc Nasopharyngeal carcinomas	0.5	–	–	0.3	–	–	1.3	–	–	0.8	–	–	–	–	–	0.5	–	–
XId Malignant melanomas	4.0	–3.7 (–5.2 to –2.1)	<.001	7.0	–0.9 (–3.8 to 2.2)	.57	0.6	–	–	1.3	–	–	–	–	–	1.4	–	–
XIe Skin carcinomas	0.1	–	–	0.1	–	–	–	–	–	–	–	–	–	–	–	–	–	–
XIf Other and unspecified carcinomas	6.4	8.8 (3.4 to 14.5)	.001	7.3	8.9 (0.8 to 17.5)	.03	5.0	–	–	4.8	–	–	8.1	–	–	5.6	10.6 (1.9 to 20.2)	.02
XII Other and unspecified malignant neoplasms	0.7	–	–	0.8	–	–	0.8	–	–	0.5	–	–	–	–	–	0.8	–	–

^a^ AAPC is the average annual percent change and is a weighted average of the APCs over the fixed interval 2008–2018 using the underlying Joinpoint model for the period of 1992–2018. AAPC was not calculated if case count was <10 cases in any 1 calendar year.

### Cancer death rates and trends

**Figure 3:**
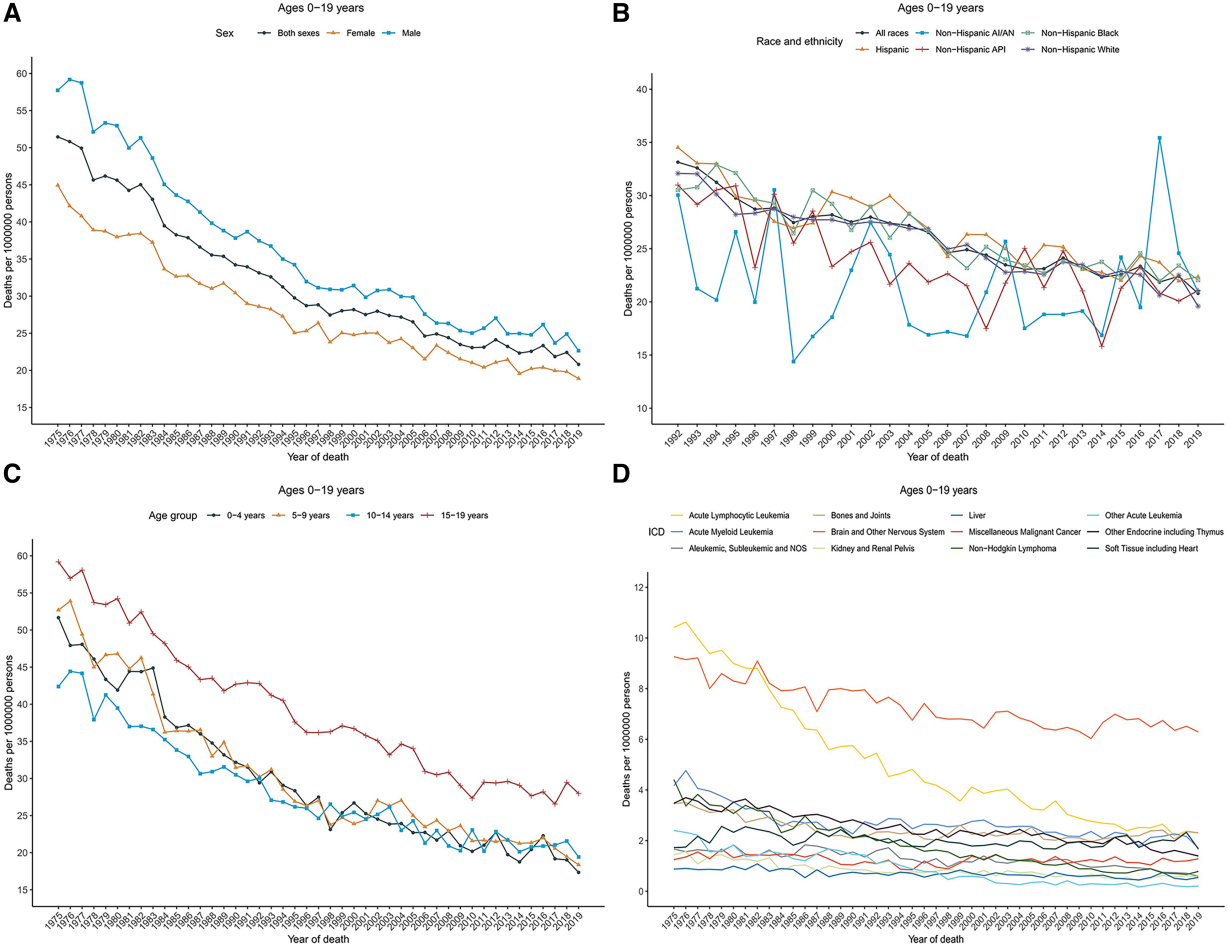
Cancer death trends for children and adolescents (ages 0–19 years) in the United States. (**A**) Annual age-adjusted death rates of all cancers by sex (1975–2019). (**B**) Cancer death rates by race and ethnicity. (**C**) Cancer death rates by age at diagnosis. (**D**) Cancer death rates by the International Classification of Diseases (ICD) group. Rates were per 1,000,000 persons, age-standardized to the 2000 US standard population (19 age groups, Census P25–1,130). AI/AN indicates American Indian and Alaska Native; and API, Asian and Pacific Islander.

Cancer death rates in patients aged 0–19 years decreased significantly over time from 51.5 in 1975 to 20.8 in 2019. Death rates decreased from 57.7 to 22.6 for males and from 44.9 to 18.9 for females during that period (Figure 3A). The overall cancer death rates have been decreasing since 1975 for both males and females. Table 2 showed the long-term trends and AAPC in 2009–2019 in death rates by age, sex, racial/ethnic group, and the top 12 common causes of death for patients aged 0–19 years. Between 1975 and 1996, the overall rates sharply decreased by 2.7% per year. From 2009 to 2019, rates decreased with an AAPC of 1.4.

**Table 2:** Joinpoint death rate trends for the ICD cancer group in children and adolescents (ages 0–19 years), United States, 1975–2019.

	Trends in 1975–2019													
	1st segment			2nd segment			3rd segment			4th segment			AAPC^a^	
	Years	APC (95% CI)	*P*	Years	APC (95% CI)	*P*	Years	APC (95% CI)	*P*	Years	APC (95% CI)	*P*	2009–2019	*P*
All sites														
Both sexes combined	1975–1996	–2.7 (–2.8 to –2.5)	<.001	1996–2019	–1.4 (–1.5 to –1.2)	<.001	–	–	–	–	–	–	–1.4 (–1.5 to –1.2)	<.001
Males	1975–1997	–2.8 (–3.0 to –2.6)	<.001	1997–2019	–1.4 (–1.6 to –1.1)	<.001	–	–	–	–	–	–	–1.4 (–1.6 to –1.1)	<.001
Females	1975–1995	–2.5 (–2.7 to –2.3)	<.001	1995–2019	–1.3 (–1.5 to –1.1)	<.001	–	–	–	–	–	–	–1.3 (–1.5 to –1.1)	<.001
Ages 0–4 years	1975–1998	–2.9 (–3.2 to –2.7)	<.001	1998–2019	–1.5 (–1.9 to –1.1)	<.001	–	–	–	–	–	–	–1.5 (–1.9 to –1.1)	<.001
Ages 5–9 years	1975–1999	–3.3 (–3.5 to –3.1)	<.001	1999–2002	3.2 (–9.4 to 17.4)	.63	2002–2019	–1.8 (–2.3 to –1.3)	<.001	–	–	–	–1.8 (–2.3 to –1.3)	<.001
Ages 10–14 years	1975–1995	–2.5 (–2.9 to –2.2)	<.001	1995–2019	–1.2 (–1.4 to –0.9)	<.001	–	–	–	–	–	–	–1.2 (–1.4 to –0.9)	<.001
Ages 0–14 years	1975–1996	–2.9 (–3.1 to –2.7)	<.001	1996–2019	–1.3 (–1.5 to –1.1)	<.001	–	–	–	–	–	–	–1.3 (–1.5 to –1.1)	<.001
Ages 15–19 years	1975–2010	–1.9 (–2.0 to –1.8)	<.001	2010–2019	–0.4 (–1.4 to 0.5)	.36	–	–	–	–	–	–	–0.6 (–1.4 to 0.2)	.16
Non-Hispanic White^b^	1992–2019	–1.5 (–1.6 to –1.3)	<.001	–	–	–	–	–	–	–	–	–	–1.5 (–1.6 to –1.3)	<.001
Non-Hispanic Black	1992–2019	–1.4 (–1.6 to –1.2)	<.001	–	–	–	–	–	–	–	–	–	–1.4 (–1.6 to –1.2)	<.001
Non-Hispanic API	1992–2019	–1.4 (–1.9 to –0.9)	<.001	–	–	–	–	–	–	–	–	–	–1.4 (–1.9 to –0.9)	<.001
Non-Hispanic AI/AN	1992–2019	–0.1 (–1.1 to 1.0)	.92	–	–	–	–	–	–	–	–	–	–0.1 (–1.1 to 1.0)	.92
Hispanic	1992–1997	–4.9 (–8.1 to –1.7)	.005	1997–2000	2.5 (–11.5 to 18.7)	.73	2000–2019	–1.6 (–1.9 to –1.2)	<.001	–	–	–	–1.6 (–1.9 to –1.2)	<.001
ICD group, both sexes														
Brain and other nervous system	1975–2001	–1.1 (–1.4 to –0.9)	<.001	2001–2019	–0.2 (–0.6 to 0.2)	.30	–	–	–	–	–	–	–0.2 (–0.6 to 0.2)	.30
Acute lymphocytic leukemia	1975–1993	–4.5 (–4.9 to –4.1)	<.001	1993–2019	–3.0 (–3.3 to –2.6)	<.001	–	–	–	–	–	–	–3.0 (–3.3 to –2.6)	<.001
Bones and joints	1975–1989	–3.2 (–4.0 to –2.4)	<.001	1989–2019	–0.1 (–0.3 to 0.2)	.67	–	–	–	–	–	–	–0.1 (–0.3 to 0.2)	.67
Acute myeloid leukemia	1975–1986	–5.1 (–6.2 to –4.0)	<.001	1986–2000	0.3 (–0.7 to 1.4)	.55	2000–2019	–1.6 (–2.2 to –1.0)	<.001	–	–	–	–1.6 (–2.2 to –1.0)	<.001
Soft tissue including heart	1975–1979	9.9 (0.8 to 19.7)	.03	1979–2001	–1.3 (–2.0 to –0.7)	<.001	2001–2019	0.6 (–0.2 to 1.4)	.16	–	–	–	0.6 (–0.2 to 1.4)	.16
Other endocrine including thymus	1975–2014	–1.7 (–1.9 to –1.5)	<.001	2014–2019	–5.4 (–9.9 to –0.8)	.02	–	–	–	–	–	–	–3.6 (–5.8 to –1.3)	.002
Miscellaneous malignant cancer	1975–1984	0.8 (–1.7 to 3.4)	.52	1984–1997	–3.6 (–5.2 to –1.9)	<.001	1997–2000	12.0 (–18.3 to 53.6)	.47	2000–2019	–0.5 (–1.3 to 0.3)	.22	–0.5 (–1.3 to 0.3)	.22
Aleukemic, sSubleukemic and NOS	1975–1993	–0.2 (–1.0 to 0.6)	.64	1993–1996	–10.0 (–30.9 to 17.3)	.42	1996–2007	0.7 (–1.4 to 2.8)	.51	2007–2019	–4.9 (–6.6 to –3.2)	<.001	–4.9 (–6.6 to –3.2)	<.001
Non-Hodgkin lymphoma	1975–2019	–4.1 (–4.3 to –3.9)	<.001	–	–	–	–	–	–	–	–	–	–4.1 (–4.3 to –3.9)	<.001
Kidney and renal [elvis	1975–1991	–3.6 (–4.7 to –2.5)	<.001	1991–2019	–1.6 (–2.2 to –1.0)	<.001	–	–	–	–	–	–	–1.6 (–2.2 to –1.0)	<.001
Liver	1975–2019	–1.2 (–1.5 to –0.9)	<.001	–	–	–	–	–	–	–	–	–	–1.2 (–1.5 to –0.9)	<.001
Other acute leukemia	1975–1981	–8.7 (–12.5 to –4.7)	<.001	1981–1990	0.8 (–2.4 to 4.1)	.60	1990–2003	–10.5 (–12.6 to –8.3)	<.001	2003–2019	–3.1 (–5.6 to –0.6)	.02	–3.1 (–5.6 to –0.6)	.02
ICD group, male														
Brain and other nervous system	1975–2009	–1.1 (–1.3 to –1.0)	<.001	2009–2012	4.9 (–10.7 to 23.2)	.55	2012–2019	–1.9 (–4.0 to 0.2)	.07	–	–	–	0.1 (–4.7 to 5.1)	.98
Acute lymphocytic leukemia	1975–1994	–4.5 (–4.9 to –4.0)	<.001	1994–2019	–3.1 (–3.5 to –2.6)	<.001	–	–	–	–	–	–	–3.1 (–3.5 to –2.6)	<.001
Bones and joints	1975–1990	–3.7 (–4.6 to –2.7)	<.001	1990–2004	1.1 (–0.3 to 2.5)	.11	2004–2007	–8.5 (–28.7 to 17.5)	.48	2007–2019	2.5 (1.0 to 4.0)	.002	2.5 (1.0 to 4.0)	.002
Acute myeloid leukemia	1975–1984	–4.7 (–6.7 to –2.7)	<.001	1984–2019	–1.0 (–1.3 to –0.7)	<.001	–	–	–	–	–	–	–1.0 (–1.3 to –0.7)	<.001
Soft tissue including heart	1975–1979	13.2 (3.0 to 24.5)	.01	1979–2005	–1.4 (–1.9 to –0.9)	<.001	2005–2019	1.3 (0.1 to 2.6)	.04	–	–	–	1.3 (0.1 to 2.6)	.04
Other endocrine including thymus	1975–2019	–1.9 (–2.0 to –1.7)	<.001	–	–	–	–	–	–	–	–	–	–1.9 (–2.0 to –1.7)	<.001
Miscellaneous malignant cancer	1975–1979	8.9 (–3.9 to 23.0)	.18	1979–1997	–3.0 (–4.4 to –1.7)	<.001	1997–2000	13.0 (–25.9 to 72.2)	.56	2000–2019	–0.9 (–2.0 to 0.2)	.10	–0.9 (–2.0 to 0.2)	.10
Non-Hodgkin lymphoma	1975–2019	–4.4 (–4.6 to –4.1)	<.001	–	–	–	–	–	–	–	–	–	–4.4 (–4.6 to –4.1)	<.001
Aleukemic, subleukemic and NOS	1975–1993	0.3 (–0.8 to 1.4)	.59	1993–1996	–12.7 (–39.6 to 26.2)	.46	1996–2007	0.6 (–2.4 to 3.6)	.70	2007–2019	–5.1 (–7.7 to –2.5)	<.001	–5.1 (–7.7 to –2.5)	<.001
Liver	1975–2019	–1.2 (–1.7 to –0.8)	<.001	–	–	–	–	–	–	–	–	–	–1.2 (–1.7 to –0.8)	<.001
Kidney and renal pelvis	1975–1990	–4.0 (–5.7 to –2.3)	<.001	1990–2019	–1.4 (–2.2 to –0.6)	.001	–	–	–	–	–	–	–1.4 (–2.2 to –0.6)	.001
Other acute leukemia	–													
ICD cancer group, female														
Brain and other nervous system	1975–1998	–1.2 (–1.5 to –0.9)	<.001	1998–2019	–0.3 (–0.7 to 0.0)	.07	–	–	–	–	–	–	–0.3 (–0.7 to 0.0)	.07
Acute lymphocytic leukemia	1975–1991	–4.6 (–5.3 to –3.8)	<.001	1991–2019	–2.8 (–3.2 to –2.4)	<.001	–	–	–	–	–	–	–2.8 (–3.2 to –2.4)	<.001
Acute myeloid leukemia	1975–1988	–5.2 (–6.4 to –3.9)	<.001	1988–1999	1.7 (–0.6 to 4.1)	.13	1999–2019	–1.7 (–2.5 to –0.9)	<.001	–	–	–	–1.7 (–2.5 to –0.9)	<.001
Bones and joints	1975–1989	–2.8 (–4.1 to –1.4)	<.001	1989–2019	–0.3 (–0.8 to 0.2)	.19	–	–	–	–	–	–	–0.3 (–0.8 to 0.2)	.19
Soft tissue including heart	1975–2019	–0.3 (–0.6 to 0.0)	.07	–	–	–	–	–	–	–	–	–	–0.3 (–0.6 to 0.0)	.07
Other endocrine including thymus	1975–2019	–1.8 (–2.0 to –1.5)	<.001	–	–	–	–	–	–	–	–	–	–1.8 (–2.0 to –1.5)	<.001
Miscellaneous malignant cancer	1975–1995	–1.7 (–2.7 to –0.7)	.002	1995–2019	0.7 (–0.1 to 1.4)	.08	–	–	–	–	–	–	0.7 (–0.1 to 1.4)	.08
Aleukemic, subleukemic and NOS	1975–2019	–1.5 (–1.8 to –1.1)	<.001	–	–	–	–	–	–	–	–	–	–1.5 (–1.8 to –1.1)	<.001
Kidney and renal pelvis	1975–2019	–2.3 (–2.7 to –2.0)	<.001	–	–	–	–	–	–	–	–	–	–2.3 (–2.7 to –2.0)	<.001
Non-Hodgkin lymphoma	1975–2019	–3.4 (–3.8 to –3.0)	<.001	–	–	–	–	–	–	–	–	–	–3.4 (–3.8 to –3.0)	<.001
Liver	1975–2019	–1.2 (–1.6 to –0.7)	<.001	–	–	–	–	–	–	–	–	–	–1.2 (–1.6 to –0.7)	<.001
Other acute leukemia	–													

^a^ AAPC is the average annual percent change and is a weighted average of the APCs over the fixed interval 2009–2019 using the underlying Joinpoint model for the period of 1975–2019.

^b^ Incidence trends by races/ethnicities were calculated between 1992 and 2019. APC, annual percent change; CI, confidence interval; AAPC, average annual percent change. APC was not calculated if case count was <10 cases in any 1 calendar year.

Overall cancer death rates in 2009–2019 were the highest among Hispanic population (23.5), followed by non-Hispanic Black (23.2), non-Hispanic White (22.4), non-Hispanic AI/AN (21.9), and non-Hispanic API (21.4) populations. However, rates for females were the highest among non-Hispanic Black (22.1), and for males were the highest among non-Hispanic AI/AN (27.4). Cancer death rates declined among non-Hispanic White [AAPC, −1.5 (95% CI, −1.6 to −1.3)], non-Hispanic Black [AAPC, −1.4 (95% CI, −1.6 to −1.2)], non-Hispanic API [AAPC, −1.4 (95% CI, −1.9 to −0.9)], and Hispanic [AAPC, −1.6 (95% CI, −1.9 to −1.2)] populations, whereas remained stable among non-Hispanic AI/AN population over the 2009–2019 period (Figure 3B).

Death rates also decreased among children [AAPC, −1.3 (95% CI, −1.5 to −1.1)] during 2009–2019, while remained stable among adolescents during that period. For children, rates decreased sharply between 1975 and 1996 [APC, −2.9 (95% CI, −3.1 to −2.7)], and then decreased relatively slowly between 1996 and 2019; for adolescents, death rates decreased 1.9% per year during 1975–2010 and then became stable between 2010 and 2019 (Figure 3C). The overall death rates in 2009–2019 were 28.5 among adolescents (aged 15–19 years), followed by 21.2 among children aged 5–9 years, 21.1 among children aged 10–14 years, and 20.2 among children aged 0–4 years.

Death rates by cause of death were represented according to the ICD nomenclature. Figure 3D displayed the cancer death rates over time categorized by cause of death according to the ICD nomenclature. Long-term trends in cancer death rates also varied by sex. During 2009–2019, death rates among males decreased for 7 of the 12 most common causes of cancer death: acute lymphocytic leukemia (ALL) with an AAPC of −3.1, AML with an AAPC of −1.0, other endocrine including thymus ((AAPC, −1.9), non-Hodgkin lymphoma (AAPC, −4.4), Aleukemic, subleukemic and NOS (AAPC, −5.1), liver (AAPC, −1.2), and kidney and renal pelvis (AAPC, −1.4); increased for 2 cancers: bones and joints (AAPC, 2.5) and soft tissue including heart (AAPC, 1.3); and were stable for 2 cancers: brain and other nervous system and miscellaneous malignant cancer. Death rates among females decreased for 7 of the 12 most common causes of cancer death: ALL (AAPC, −2.8), AML (AAPC, −1.7), other endocrine including thymus ((AAPC, −1.8), non-Hodgkin lymphoma (AAPC, −3.4), Aleukemic, subleukemic and NOS (AAPC, −1.5), liver (AAPC, −1.2), and kidney and renal pelvis (AAPC, −2.3), and were stable for 4 cancers: bones and joints, soft tissue including heart, brain and other nervous system, and miscellaneous malignant cancer. Overall death rates in 2009–2019 by the top 12 common causes of death for children, adolescents, and children and adolescents were displayed in Supplementary Figure 1. Brain and other nervous system (rate, 6.9), ALL (2.4), and other endocrine including thymus (2.1) ranked as the top three cancers with the highest death rates among children, whereas brain and other nervous system (5.3), bones and joints (4.9), and soft tissue including heart (3.1) were the most common causes of death among adolescents.

Supplementary Table S2 presented the death rates and trends in 2009–2019 by cause of death for non-Hispanic White, non-Hispanic Black, and Hispanic populations. Notably, some of the rates and trends results could not be calculated due to the limited number of cases. During 2009–2019, among non-Hispanic White persons, death rates decreased for most of the common cancers except for bones and joints, soft tissue including heart, and miscellaneous malignant cancer, death rates for which remained stable during that period. Trends in death rates for brain and other nervous system, bones and joints, and soft tissue including heart were stable among non-Hispanic Black persons. Trends in death rates for brain and other nervous system, bones and joints, soft tissue including heart, miscellaneous malignant cancer were table among Hispanic population.

## Discussion

The incidence rates of cancer in individuals aged 0 to 19 years in the United States has increased 0.8% per year since 1975 (14). The reasons behind this increasing trend might include changes in environmental factors, improved diagnosis, and improved access to medical care (7, 15). The incidence rates of cancer increased at different rates among children and adolescents between 2008 and 2018, with AAPCs of 0.8 and 1.3 respectively. Specifically, the rise in childhood cancer was characterized by the increase in children aged 10 to 14 years, among whom the incidence rates increased at an average annual rate of 1.8%, which differed slightly from another study reporting that incidence rate of cancer stabilized in children but continued to increase in adolescents during 2009 to 2019 (4). Notably, previous studies came to different conclusions that the cancer incidence rates among children averagely increased 0.7% per year during 2013–2017 but remained stable during 2014–2018 (13, 16). The variations in these findings could potentially be attributed to disparities in the time period examined and the extent of population coverage (6). With respect to cancer mortality rates, the death rates decreased among children since 1975 but stabilized in adolescents during 2009 through 2019 after increasing since 1975. The lower and even stagnant reductions in cancer mortality among adolescents compared to children may be attributed to the slower progress for certain common cancers in adolescents, such as acute lymphoblastic leukemia, non-Hodgkin lymphoma, and brain and other nervous system tumors, partly because of variances in tumor biology, treatment protocols and so on (4, 17).

Cancer incidence and mortality rates varied by race and ethnicity. Non-Hispanic White and Hispanic children exhibited the highest overall incidence rates among childhood and adolescent cancers during the specified time period, while non-Hispanic Black and non-Hispanic AI/AN children have similar and the lowest incidence rates. Hispanic and non-Hispanic Black children have the highest cancer mortality rates, when stratified by sex, non-Hispanic Black girls and non-Hispanic AI/AN boys have the highest cancer death rates. The underlying causes for differences in the cancer mortality of childhood cancers among different racial and ethnic groups in the United States remained elusive. Potential factors such as socioeconomic status, health insurance coverage, delay in diagnosis, differences in drug metabolism, and so on, which were associated with disparities in survival, might partly explain the differences in the cancer mortality (18–20). Previous reports demonstrated that childhood cancer survivors from non-Hispanic Black and Hispanic backgrounds, when compared to non-Hispanic White individuals, experience greater challenges and poorer health outcomes (21). Of note was the health insurance coverage, which accounted for 20% and 48% of the survival disparities between non-Hispanic Black and Hispanic children and adolescents with cancer compared to their non-Hispanic White counterparts.

The ICCC-3 classification system was used to report cancer incidence rates in children and adolescents (2, 15). The most common cancers in this age group were leukemias, CNS tumors, and lymphomas. During 2008–2018, incidence rates for leukemias increased both among boys and girls of all racial and ethnic groups except for non-Hispanic AI/AN, incidence trends for which could not be calculated because of few cases. Specifically, incidence rates of lymphoid leukemias increased among non-Hispanic White, non-Hispanic API, and Hispanic children, whereas incidence rates of AML increased among non-Hispanic Black children. It has been reported that there is a notable peak in the incidence rates of ALL among children at the ages of 2 and 4 years in industrialized countries, which is particularly pronounced in White and Hispanic children compared with Black children in the United States (7, 22). In contrast to the rise in incidence, mortality rates in this age group decreased for ALL, AML, and other acute leukemia during 2009–2019, as previous studies reported that over the past 4 decades, the refinement of chemotherapy regimens for childhood ALL, utilizing improved combinations of predominantly the same agents, has achieved impressive remission rates of 90% to 100% (23). However, mortality rates for those leukemias, especially for ALL, were higher among adolescents compared with children, which could be partly attributed to the inferior prognosis of adolescents with ALL compared with younger children, as adolescent ALL is a disease entity distinct from its pediatric and adult counterparts (17).

Incidence rates of CNS tumors increased among children and adolescents during 2008–2018, driven by a rise in girls rather than boys. Consistent with previous findings, the most common subtype of CNS tumor was astrocytoma, with the highest incidence rates observed in non-Hispanic White individuals (7). Additionally, the incidence rates of astrocytoma continued to increase within this racial and ethnic group. While the incidence rates were highest in non-Hispanic White individuals, mortality rates for brain and other nervous system in non-Hispanic White and non-Hispanic Black children were similar (24). In addition, the death rates decreased in non-Hispanic White but remained stable in non-Hispanic Black and Hispanic children from 2009 through 2019. It was reported that non-Hispanic Black and Hispanic children diagnosed with malignant tumors had lower survival compared with their White counterparts, which could be partly attributed to limited access to optimal healthcare within these populations (25).

Notable increasing trends in hepatic cancer incidence rates have been observed in this age group since 1975, consistent with previous findings, although the absolute incidence rates remain relatively low (26, 27). Hepatoblastoma is the most common subtype of malignant liver tumor in children. The increasing incidence of hepatic tumor was predominated by rise in incidence of hepatoblastoma, with the most significant increase observed among males and in children aged 2–4 years old (27, 28). The decreasing mortality rates of liver cancer were observed both in males and females. This decrease could potentially be associated with the prolonged overall survival in children with hepatoblastoma, which was attributed to advancements in treatment (28).

As noted in previous findings, the incidence rates of pediatric thyroid cancer have been increasing since the 1970s (29). The increasing trends persisted across all racial and ethnic groups, with the highest rate found in non-Hispanic White and the lowest rate in non-Hispanic Black children. A study demonstrated that a sharp increase in incidence rate occurred after 2006, which could be partly attributed to the guidelines published by the American Thyroid Association in 2006 potentially leading to enhanced detection through improved diagnostic ultrasonography and ultrasonography-guided biopsies (10). In combination with the overdiagnosis, the actual increase in childhood thyroid cancer incidence rate, which might be associated with environmental exposures, in particular, exposure to ionizing radiation, could together contribute to the increasing trends in incidence of pediatric thyroid cancer (10, 30–32).

There were several limitations to this study. The SEER database, while comprehensive within the United States, is regionally selective and may not fully represent all geographic or demographic groups within the country. This might affect the extrapolation of our findings to states or areas not covered by the SEER program. Further studies might be necessary to confirm these trends in states or regions not represented in the SEER data. In addition, using metrics like cancer incidence and mortality rates could not thoroughly estimate the disease burden of childhood cancer given that the long-term effects of cancer treatments characterized by survivorship morbidity were overlooked (3). In addition, there is a lack of data pertaining to minor racial and ethnic groups, which may result in underestimates of cancer in these subpopulations. Finally, the joinpoint regression analysis used in our study, although powerful for identifying changes in trends, has limitations. The method assumes that changes in trends are best described by piecewise linear segments, which may not capture more complex patterns in the data. Moreover, the selection of too many or too few joinpoints can lead to overfitting or underfitting the model to the data, respectively.

In summary, we examined the cancer incidence and mortality rates and trends in children aged 0–19 years in the United States. Our findings demonstrated that the overall cancer incidence rates increased among children and adolescents, while the cancer mortality rates declined overall and for many cancer types over time. Rates and trends varied by age, sex, and especially race and ethnicity, underscoring the importance of understanding and addressing disparities and finally reducing the disease burden of childhood and adolescent cancer.

## Data Availability

The raw data supporting the conclusions of this article will be made available by the authors, without undue reservation.
