# Supplementary material for: Long-term trends in cancer incidence and mortality among U.S. children and adolescents: a SEER database analysis from 1975 to 2018
Source: Front Pediatr. 2024 Jul 5;12:1357093. doi: 10.3389/fped.2024.1357093 (PMC11258622; doi:10.3389/fped.2024.1357093)
Supplement: Supplementary file 1 [file Datasheet1.pdf]

Supplementary Table 1. Joinpoint incidence trends for the ICCC-3 cancer group in children and adolescents (Ages 0-19 years), United States, 1975-2018.

| | Trends in 1975-2018 | | | | | | | | | | | | | |
|---|---|---|---|---|---|---|---|---|---|---|---|---|---|---|
| | 1st segment | | | 2nd segment | | | 3rd segment | | | 4th segment | | | AAPC[a] | |
| | Years | APC (95% CI) | *P* | Years | APC (95% CI) | *P* | Years | APC (95% CI) | *P* | Years | APC (95% CI) | *P* | 2008-2018 | *P* |
| All sites | | | | | | | | | | | | | | |
| Both sexes combined | 1975-2018 | 0.8 (0.7 to 0.9) | < .001 | – | – | – | – | – | – | – | – | – | 0.8 (0.7 to 0.9) | < .001 |
| Males | 1975-2018 | 0.8 (0.7 to 0.9) | < .001 | – | – | – | – | – | – | – | – | – | 0.8 (0.7 to 0.9) | < .001 |
| Females | 1975-2006 | 0.6 (0.4 to 0.7) | < .001 | 2006-2015 | 1.8 (1.0 to 2.7) | < .001 | 2015-2018 | –2.0 (–5.5 to 1.7) | .27 | – | – | – | 0.7 (–0.5 to 1.9) | .28 |
| Ages 0-4 years | 1975-2015 | 0.8 (0.7 to 0.9) | < .001 | 2015-2018 | –2.9 (–6.8 to 1.2) | .16 | – | – | – | – | – | – | –0.3 (–1.5 to 0.9) | .60 |
| Ages 5-9 years | 1975-2006 | 0.5 (0.2 to 0.7) | .001 | 2006-2009 | 4.7 (–6.7 to 17.4) | .43 | 2009-2018 | – 0.0 (–1.0 to 1.0) | .94 | – | – | – | 0.4 (–1.0 to 1.9) | .56 |

| | | | | | | | | | | | | | | |
|---|---|---|---|---|---|---|---|---|---|---|---|---|---|---|
| Ages 10-14 years | 1975-2006 | 0.7 (0.5 to 1.0) | < .001 | 2006-2018 | 1.8 (1.1 to 2.4) | < .001 | – | – | – | – | – | – | 1.8 (1.1 to 2.4) | < .001 |
| Ages 0-14 years | 1975-2018 | 0.8 (0.7 to 0.9) | < .001 | – | – | – | – | – | – | – | – | – | 0.8 (0.7 to 0.9) | < .001 |
| Ages 15-19 years | 1975-2006 | 0.5 (0.3 to 0.7) | < .001 | 2006-2018 | 1.3 (0.8 to 1.8) | < .001 | – | – | – | – | – | – | 1.3 (0.8 to 1.8) | < .001 |
| Non-Hispanic White[b] | 1992-2018 | 0.9 (0.7 to 1.1) | < .001 | – | – | – | – | – | – | – | – | – | 0.9 (0.7 to 1.1) | < .001 |
| Non-Hispanic Black | 1992-1998 | –2.6 (–6.0 to 0.9) | .13 | 1998-2013 | 2.0 (1.4 to 2.7) | < .001 | 2013-2018 | –1.6 (–4.3 to 1.2) | .25 | – | – | – | 0.2 (–1.1 to 1.6) | .75 |
| Non-Hispanic API | 1992-2004 | –0.4 (–1.7 to 1.0) | .56 | 2004-2018 | 2.0 (1.2 to 2.8) | < .001 | – | – | – | – | – | – | 2.0 (1.2 to 2.8) | < .001 |
| Non-Hispanic AI/AN | 1992-2018 | 1.4 (0.7 to 2.2) | < .001 | – | – | – | – | – | – | – | – | – | 1.4 (0.7 to 2.2) | < .001 |
| Hispanic | 1992-2018 | 1.0 (0.8 to 1.3) | < .001 | – | – | – | – | – | – | – | – | – | 1.0 (0.8 to 1.3) | < .001 |
| ICCC-3 cancer group, both sexes | | | | | | | | | | | | | | |

| | | | | | | | | | | | | | | |
|---|---|---|---|---|---|---|---|---|---|---|---|---|---|---|
| I Leukemias, myeloproliferative diseases, and myelodysplastic diseases | 1975-2009 | 1.2 (1.0 to 1.4) | < .001 | 2009-2018 | 0.3 (–0.4 to 1.1) | .36 | – | – | – | – | – | – | 0.4 (–0.2 to 1.1) | .20 |
| II Lymphomas and reticuloendothelial neoplasms | 1975-2004 | –0.3 (–0.5 to 0.0) | .06 | 2004-2015 | 3.2 (2.3 to 4.1) | < .001 | 2015-2018 | –2.6 (–7.8 to 2.9) | .33 | – | – | – | 1.4 (–0.3 to 3.2) | .10 |
| III CNS and miscellaneous intracranial and intraspinal neoplasms | 1975-1988 | 2.1 (0.6 to 3.7) | .008 | 1988-2018 | 0.3 (0.0 to 0.6) | .03 | – | – | – | – | – | – | 0.3 (0.0 to 0.6) | .037 |
| IV Neuroblastoma, other peripheral nervous cell tumors | 1975-2018 | –0.0 (–0.3 to 0.3) | .82 | – | – | – | – | – | – | – | – | – | –0.0 (–0.3 to 0.3) | .82 |
| V Retinoblastoma | 1975-2018 | 0.3 (–0.0 to 0.6) | .07 | – | – | – | – | – | – | – | – | – | 0.3 (–0.0 to 0.6) | .07 |
| VI Renal tumors | 1975-2018 | 0.1 (–0.1 to 0.4) | .30 | – | – | – | – | – | – | – | – | – | 0.1 (–0.1 to 0.4) | .30 |
| VII Hepatic tumors | 1975-2018 | 1.9 (1.3 to 2.4) | < .001 | – | – | – | – | – | – | – | – | – | 1.9 (1.3 to 2.4) | < .001 |
| VIII Malignant bone tumors | 1975-2018 | 0.3 (0.0 to 0.6) | .03 | – | – | – | – | – | – | – | – | – | 0.3 (0.0 to | .03 |

| | | | | | | | | | | | | | | |
|---|---|---|---|---|---|---|---|---|---|---|---|---|---|---|
| | | | | | | | | | | | | | 0.6) | |
| IX Soft tissue and other extraosseous sarcomas | 1975-2018 | 0.4 (0.1 to 0.6) | .002 | – | – | – | – | – | – | – | – | – | 0.4 (0.1 to 0.6) | .002 |
| X Germ cell tumors, trophoblastic tumors, and neoplasms of gonads | 1975-2018 | 0.7 (0.4 to 0.9) | < .001 | – | – | – | – | – | – | – | – | – | 0.7 (0.4 to 0.9) | < .001 |
| XI Other malignant epithelial neoplasms and malignant melanomas | 1975-2008 | 0.8 (0.4 to 1.1) | < .001 | 2008-2018 | 4.6 (3.4 to 5.8) | < .001 | – | – | – | – | – | – | 4.6 (3.4 to 5.8) | < .001 |
| XII Other and unspecified malignant neoplasms | 1975-2018 | –0.2 (–1.0 to 0.6) | .68 | – | – | – | – | – | – | – | – | – | –0.2 (–1.0 to 0.6) | .68 |
| ICCC-3 cancer group, male | | | | | | | | | | | | | | |
| I Leukemias, myeloproliferative diseases, and myelodysplastic diseases | 1975-2018 | 1.0 (0.9 to 1.2) | < .001 | – | – | – | – | – | – | – | – | – | 1.0 (0.9 to 1.2) | < .001 |
| II Lymphomas and reticuloendothelial neoplasms | 1975-2004 | –0.3 (–0.7 to 0.0) | .08 | 2004-2014 | 3.9 (2.5 to 5.3) | < .001 | 2014-2018 | –1.9 (–6.1 to 2.5) | .39 | – | – | – | 1.6 (–0.3 to 3.5) | .10 |

| | | | | | | | | | | | | | | |
|---|---|---|---|---|---|---|---|---|---|---|---|---|---|---|
| III CNS and miscellaneous intracranial and intraspinal neoplasms | 1975-1988 | 2.4 (0.6 to 4.2) | .008 | 1988-2018 | 0.1 (–0.2 to 0.4) | .39 | – | – | – | – | – | – | 0.1 (–0.2 to 0.4) | .39 |
| IV Neuroblastoma, other peripheral nervous cell tumors | 1975-2018 | –0.1 (–0.5 to 0.3) | .64 | – | – | – | – | – | – | – | – | – | –0.1 (–0.5 to 0.3) | .64 |
| V Retinoblastoma | 1975-2018 | 0.3 (–0.3 to 0.9) | .34 | – | – | – | – | – | – | – | – | – | 0.3 (–0.3 to 0.9) | .34 |
| VI Renal tumors | 1975-2018 | 0.1 (–0.3 to 0.4) | .73 | – | – | – | – | – | – | – | – | – | 0.1 (–0.3 to 0.4) | .73 |
| VII Hepatic tumors | 1975-2018 | 2.1 (1.3 to 2.8) | < .001 | – | – | – | – | – | – | – | – | – | 2.1 (1.3 to 2.8) | < .001 |
| VIII Malignant bone tumors | 1975-2018 | 0.4 (0.0 to 0.8) | .05 | – | – | – | – | – | – | – | – | – | 0.4 (0.0 to 0.8) | .05 |
| IX Soft tissue and other extraosseous sarcomas | 1975-2018 | 0.4 (0.1 to 0.7) | .008 | – | – | – | – | – | – | – | – | – | 0.4 (0.1 to 0.7) | .008 |
| X Germ cell tumors, trophoblastic tumors, and neoplasms of gonads | 1975-2018 | 1.0 (0.6 to 1.3) | < .001 | – | – | – | – | – | – | – | – | – | 1.0 (0.6 to 1.3) | < .001 |

| | | | | | | | | | | | | | | |
|---|---|---|---|---|---|---|---|---|---|---|---|---|---|---|
| XI Other malignant epithelial neoplasms and malignant melanomas | 1975-2011 | 0.9 (0.4 to 1.3) | < .001 | 2011-2018 | 4.6 (1.5 to 7.8) | .004 | – | – | – | – | – | – | 3.5 (1.4 to 5.6) | .001 |
| XII Other and unspecified malignant neoplasms | – | | | | | | | | | | | | | |
| ICCC-3 cancer group, female | | | | | | | | | | | | | | |
| I Leukemias, myeloproliferative diseases, and myelodysplastic diseases | 1975-2018 | 1.1 (0.9 to 1.3) | < .001 | – | – | – | – | – | – | – | – | – | 1.1 (0.9 to 1.3) | < .001 |
| II Lymphomas and reticuloendothelial neoplasms | 1975-2003 | –0.2 (–0.7 to 0.2) | .24 | 2003-2018 | 2.0 (1.3 to 2.7) | < .001 | – | – | – | – | – | – | 2.0 (1.3 to 2.7) | < .001 |
| III CNS and miscellaneous intracranial and intraspinal neoplasms | 1975-2018 | 0.7 (0.4 to 1.0) | < .001 | – | – | – | – | – | – | – | – | – | 0.7 (0.4 to 1.0) | < .001 |
| IV Neuroblastoma, other peripheral nervous cell tumors | 1975-2018 | – 0.0 (–0.4 to 0.4) | .83 | – | – | – | – | – | – | – | – | – | 0.0 (–0.4 to 0.4) | .83 |
| V Retinoblastoma | 1975-2018 | 0.1 (–0.4 to 0.7) | .62 | – | – | – | – | – | – | – | – | – | 0.1 (–0.4 to 0.7) | .62 |

| | | | | | | | | | | | | | | |
|---|---|---|---|---|---|---|---|---|---|---|---|---|---|---|
| VI Renal tumors | 1975-2018 | 0.2 (–0.2 to 0.6) | .31 | – | – | – | – | – | – | – | – | – | 0.2 (–0.2 to 0.6) | .31 |
| VII Hepatic tumors | 1975-2018 | 1.4 (0.6 to 2.3) | .001 | – | – | – | – | – | – | – | – | – | 1.4 (0.6 to 2.3) | .001 |
| VIII Malignant bone tumors | 1975-2018 | 0.1 (–0.3 to 0.6) | .48 | – | – | – | – | – | – | – | – | – | 0.1 (–0.3 to 0.6) | .48 |
| IX Soft tissue and other extraosseous sarcomas | 1975-2018 | 0.3 (0.0 to 0.7) | .05 | – | – | – | – | – | – | – | – | – | 0.3 (0.0 to 0.7) | .05 |
| X Germ cell tumors, trophoblastic tumors, and neoplasms of gonads | 1975-2018 | 0.2 (–0.2 to 0.5) | .33 | – | – | – | – | – | – | – | – | – | 0.2 (–0.2 to 0.5) | .33 |
| XI Other malignant epithelial neoplasms and malignant melanomas | 1975-2006 | 0.6 (0.2 to 1.0) | .008 | 2006-2018 | 4.5 (3.5 to 5.6) | < .001 | – | – | – | – | – | – | 4.5 (3.5 to 5.6) | < .001 |
| XII Other and unspecified malignant neoplasms | – | | | | | | | | | | | | | |

[a] AAPC is the average annual percent change and is a weighted average of the APCs over the fixed interval 2008-2018 using the underlying Joinpoint model for the period of 1975-2018.

[b] Incidence trends by races/ethnicities were calculated between 1992 and 2018. APC, annual percent change; CI, confidence interval; AAPC, average annual percent change. APC was not calculated if case count was <10 cases in any 1 calendar year.

Supplementary Table 2. Age-standardized death rates and fixed-interval trends (2009-2019) for the ICD cancer group by racial/ethnic group, ages 0-19 years.

| | Non–Hispanic White | | | Non–Hispanic Black | | | Hispanic | | |
|---|---|---|---|---|---|---|---|---|---|
| ICD cancer group | Rate | AAPC (95% CI) | P | Rate | AAPC (95% CI) | P | Rate | AAPC (95% CI) | P |
| Brain and other nervous system | 6.8 | –0.4 (–0.6 to –0.1) | .006 | 6.7 | –0.1 (–0.7 to 0.4) | .63 | 6.0 | –0.3 (–0.7 to 0.2) | .26 |
| Acute lymphocytic leukemia | 2.1 | –3.5 (–3.9 to –3.1) | < .001 | 1.7 | –3.2 (–4.3 to –2.2) | < .001 | 4.1 | –2.9 (–3.4 to –2.3) | < .001 |
| Bones and joints | 2.2 | 0.0 (–0.5 to 0.5) | .89 | 1.9 | –0.6 (–1.4 to 0.1) | .10 | 2.3 | –0.5 (–1.2 to 0.2) | .16 |
| Acute myeloid leukemia | 1.9 | –1.5 (–2.0 to –1.0) | < .001 | 2.2 | –1.3 (–2.2 to –0.4) | .008 | 2.4 | –0.9 (–1.6 to –0.1) | .02 |
| Soft tissue including heart | 2.0 | 0.4 (–0.0 to 0.8) | .08 | 2.2 | –0.1 (–1.0 to 0.9) | .91 | 1.8 | 0.3 (–0.5 to 1.2) | .40 |
| Other endocrine including thymus | 2.1 | –1.3 (–1.8 to –0.8) | < .001 | 2.0 | –5.0 (–8.5 to –1.5) | .008 | 1.1 | –2.1 (–3.4 to –0.7) | .004 |
| Miscellaneous malignant cancer | 1.2 | 0.6 (–0.2 to 1.3) | .12 | 1.1 | – | | 1.2 | 0.5 (–0.8 to 1.8) | .45 |

| | | | | | | | | | |
|---|---|---|---|---|---|---|---|---|---|
| Aleukemic, subleukemic and NOS | 0.8 | –2.7 (–3.4 to –1.9) | < .001 | 0.8 | – | | 1.0 | –5.4 (–8.2 to –2.5) | .001 |
| Non–Hodgkin lymphoma | 0.8 | –4.3 (–4.9 to –3.7) | < .001 | 1.1 | –2.0 (–3.0 to –0.9) | .001 | 0.7 | – | |
| Kidney and renal pelvis | 0.5 | –4.3 (–6.2 to –2.2) | < .001 | 1.0 | – | | 0.5 | – | |
| Liver | 0.5 | –1.2 (–2.1 to –0.4) | .006 | 0.6 | – | | 0.6 | – | |
| Other acute leukemia | 0.2 | – | | 0.3 | – | | 0.3 | – | |

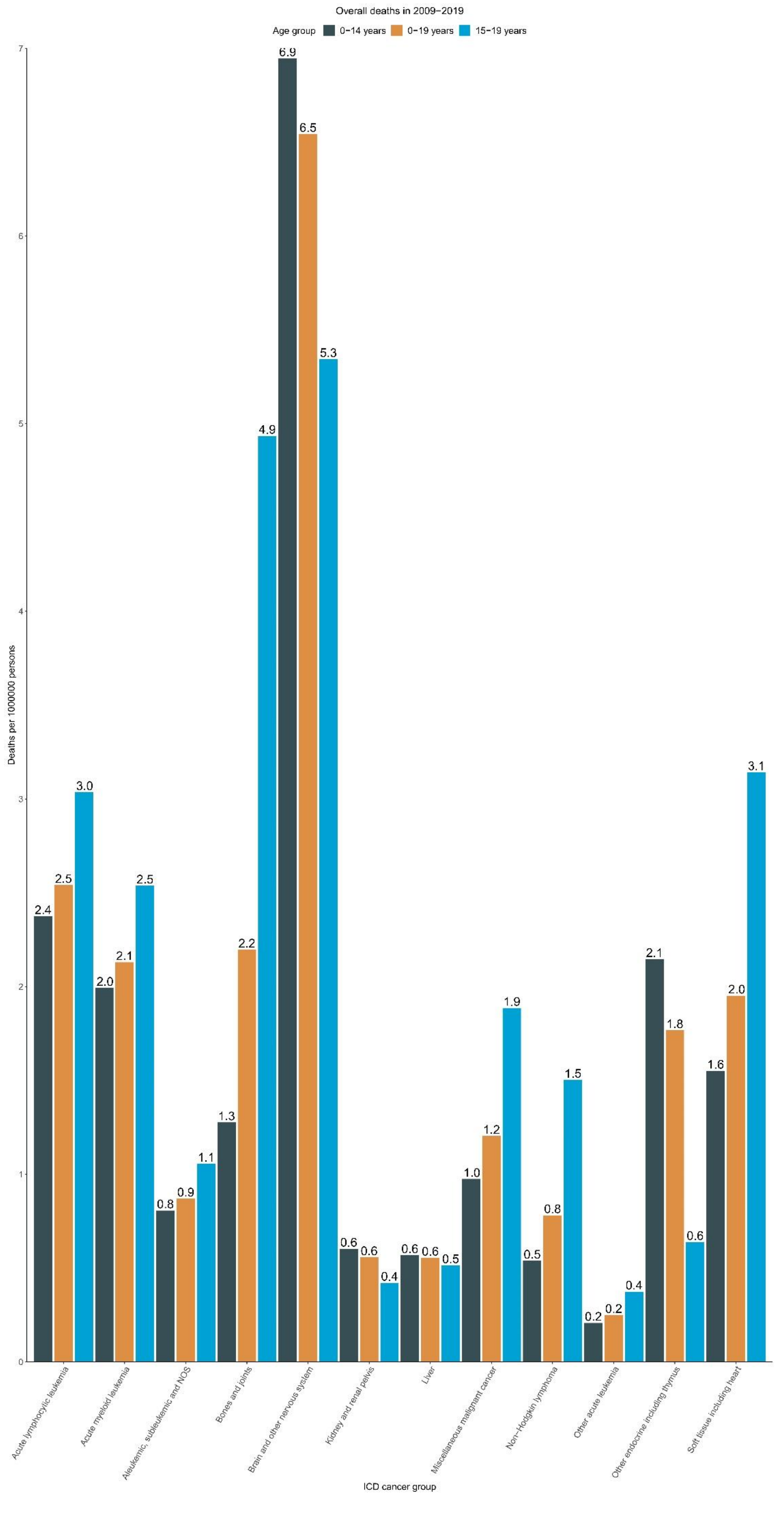

Overall deaths in 2009−2019
Age group
0−14 years
0−19 years
15−19 years
Deaths per 1000000 persons
0
1
2
3
4
5
6
7
ICD cancer group
Acute lymphocytic leukemia
Acute myeloid leukemia
Aleukemic, subleukemic and NOS
Bones and joints
Brain and other nervous system
Kidney and renal pelvis
Liver
Miscellaneous malignant cancer
Non−Hodgkin lymphoma
Other acute leukemia
Other endocrine including thymus
Soft tissue including heart
2.4
2.5
3.0
2.0
2.1
2.5
0.8
0.9
1.1
1.3
2.2
4.9
6.9
6.5
5.3
0.6
0.6
0.4
0.6
0.6
0.5
1.0
1.2
1.9
0.5
0.8
1.5
0.2
0.2
0.4
2.1
1.8
0.6
1.6
2.0
3.1

Supplementary Figure 1. Cancer death rates of children, adolescents, and children and adolescents in the United States, 2009–2019, by the International Classification of Disease (ICD) group. Rates were per 1000000 persons, age-standardized to the 2000 US standard population (19 age groups, Census P25–1130).
